# A rare coronary anomaly consisting of a single right coronary ostium in an adult undergoing surgical coronary revascularization: a case report and review of the literature

**DOI:** 10.1186/s13256-016-0977-5

**Published:** 2016-07-01

**Authors:** Edvin Prifti, Fadil Ademaj, Klodian Krakulli, Edlira Rruci, Merita Zeka, Aurel Demiraj

**Affiliations:** 1 Division of Cardiac Surgery, University Hospital Center of Tirana, Tirana, Albania; 2 Division of Cardiology, Gjakovo Hospital, Rr. Prizren, Gjakovo, Kosovo

**Keywords:** Case report, Single coronary ostium, Anomalous origin of the coronary arteries, T-coronary vessel

## Abstract

**Background:**

Coronary arteries originating from the right coronary ostium in the ascending aorta represent a very rare anatomic presentation. Also, the presence of a single coronary ostium is an extremely rare finding.

**Case presentation:**

We present the case of a 74-year-old Albanian man from Kosovo. He had unstable angina due to critical triple vessel disease and a single coronary artery originating from a single ostium in the right sinus of Valsalva with an anomalous course of his left anterior descending artery anteriorly to the right ventricular outflow tract as a “T-vessel” from which originated the proximal and distal left anterior descending artery, the circumflex artery originating from the mid portion of the right coronary artery which had a normal course. He underwent successful coronary revascularization consisting of three vein grafts to the right coronary artery, first diagonal and obtuse marginal artery, and left internal mammary artery anastomosed to left anterior descending artery.

**Conclusions:**

We describe a proposed IID1 pattern. After a careful revision of the literature, only six cases have been reported with a similar anomalous coronary origin. Only two out of six patients underwent surgical coronary revascularization. In our case the aberrant vessel arising from his right coronary artery coursed anteriorly to the right ventricle and continued as a left anterior descending artery at its mid portion which then continued distally as the distal left anterior descending artery and proximally as a proximal left anterior descending artery, having the shape of a “T vessel”. The “T-vessel” configuration has never been reported in the literature.

The reported case with its specific presentation adds further information on this rare form of anomalous origin of the coronary arteries, representing a first report of a configuration that we name the “T-vessel” of the left anterior descending artery. Diagnosis of the coronary anatomy is very important for the invasive cardiologist and cardiac surgeon in cases with a single coronary ostium, such as our case, so that they can proceed with the invasive or surgical treatment when critical coronary artery disease is present.

## Background

Coronary arteries originating from a single coronary ostium are very rare [1], occurring in less than 0.02 to 0.06 % of the general population [2]. Classifications have evolved as different anatomical variations were reported either angiographically or as necropsy findings [1]. We present a case with triple vessel disease and all three coronary arteries originating from a single ostium in the right sinus of Valsalva with an anomalous course of the left anterior descending artery anterior to the right ventricular outflow tract trunk as a “T-vessel”, and circumflex artery originating from the mid portion of the right coronary artery which had a normal course. The reported case represents the first reported configuration of the coronary arteries anatomy that we name the “T-vessel” of the left anterior descending artery. Diagnosis of the coronary anatomy is very important for the invasive cardiologist and cardiac surgeon in cases with a single coronary ostium, so that they can proceed successfully with invasive or surgical treatment.

## Case presentation

**Fig. 1:**
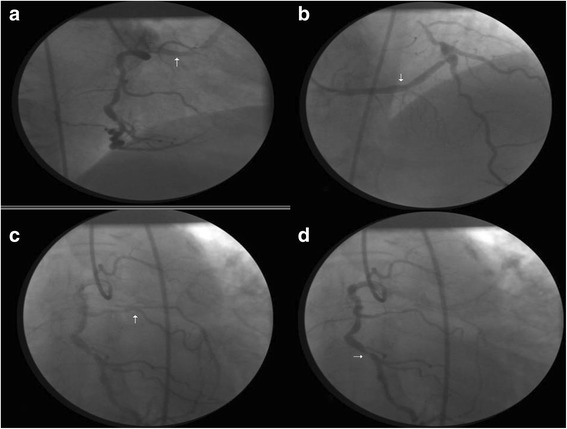
Coronary angiography demonstrating the **a** right ostium and the aberrant vessel originating closed to the ostium (arrow) and **b** then coursing anteriorly to the right ventricle from which originates the left anterior descending artery as a “T-vessel” (arrow). **c** The origin of the obtuse marginal artery from the mid portion of the right coronary artery (arrow). **d** The critical stenotic lesion at the origin of the posterior descending artery (arrow)

**Fig. 2:**
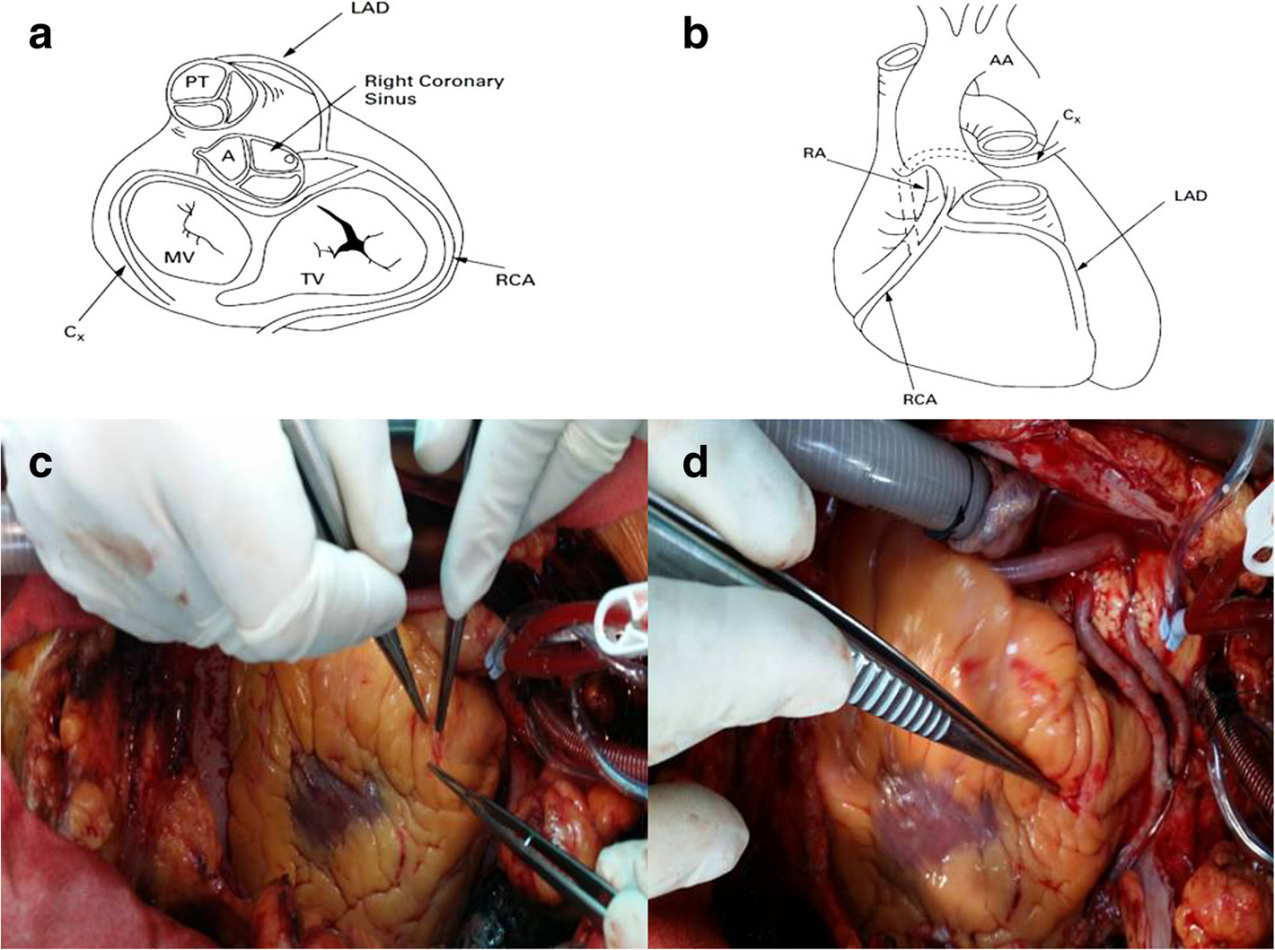
**a** Diagrammatic representation of the course of the coronary arteries from above in relation to the cardiac valve. **b** The course of the circumflex artery. **c** Intraoperative visualization of the aberrant vessel coursing anteriorly the right ventricle. **d** The final intraoperative view of the coronary revascularization procedure. *AA *Aortic Arch, *C*_*x*_ circumflex artery, *LAD* left anterior descending artery, *RCA* right coronary artery, *RA *right atrium, *PT *Pulmonary trunk, *A *Aorta, *TV *Tricuspid Valve, *MV *Mitral Valve

A 74-year-old Albanian man from Kosovo presented to our cardiology department because of unstable angina. The associated risk factors included diabetes, tobacco smoking, and hypercholesterolemia. Four months earlier, he had experienced a lateral wall myocardial infarction which was not treated percutaneously because he was admitted 3 days after the onset of myocardial infarction. His left ventricular ejection fraction was 52 %. He underwent coronary angiography. We were unable to selectively cannulate his left coronary artery, and a nonselective injection disclosed no coronary artery arising from the left coronary sinus. On selective injection of his right coronary sinus, all three coronary arteries originated from a single ostium in the right coronary sinus. Close to the right coronary ostium originated an aberrant large vessel (Fig. 1a) which coursed transversely and anterior to the right ventricular outflow tract (Fig. 2a), from which the left anterior descending artery arose and coursed to the anterior interventricular groove continuing distally and proximally as a normal left anterior descending artery, it had the shape of a “T-vessel”, presenting a critical stenosis at the bifurcation of the first diagonal artery (Fig. 1b). Another aberrant vessel arose at the mid portion of his right coronary artery, coursed to the left, behind the aorta, and in front of the atria to reach the atrioventricular groove on the left and then travelled the normal course with an important obtuse marginal artery (Figs. 1c and 2b) presenting a critical proximal stenosis. His right coronary artery followed a normal course presenting a critical stenosis at the origin of the posterior descending artery (Fig. 1d).

He underwent coronary artery bypass grafting. His heart was arrested employing intermittent anterior cold blood cardioplegia. The aortic cross clamping time was 57 minutes and extracorporeal circulation was 70 minutes. Intraoperatively the aberrant vessel from which the left anterior descending artery originated was easily found (Fig. 2c). The coronary revascularization consisted in left internal mammary artery anastomosed to the left anterior descending artery, and vein graft to the first diagonal artery, obtuse marginal artery and posterior descending artery (Fig. 2d). His postoperative course was uneventful.

## Discussion

The coronary circulation arising from a single coronary ostium has little clinical significance, except for cases in which a coronary artery traverses between the pulmonary artery and aorta, which can cause sudden death at a young age due to extrinsic coronary arterial occlusion [1]. The other clinical implications involve difficulty visualizing the circulation angiographically and accidental damage to an aberrant coursing artery during cardiac surgery [3].

Anomalous coronary arteries are associated with ischemia and sudden death, which could be the result of compression by the aorta and pulmonary artery. However, ischemia is also reported when an anomalous coronary artery does not run between the great vessels as in our case [4]. Our patient has a solitary ostium originating from the right sinus of Valsalva with his left anterior descending artery, anterior to the right ventricular outflow tract and the circumflex artery following its course retroaortically. The origin of all three coronary arteries from the right coronary sinus with separated ostia, associated with a course of the left anterior descending artery anterior to the right ventricular outflow tract is very rarely reported [5]. All three coronary arteries originating from a single ostium from the right coronary sinus with a similar course of the left anterior descending artery is extremely rare [1]. The classification by Shirani and Roberts [1] is anatomically based, depending on the location of the coronary ostium, coronary sinus in which the ostium is located, and the pattern of distribution of the coronary arteries. In this classification, all possible anatomical variations were considered, whether or not a case has been reported. We describe a proposed IID1 pattern: a solitary ostium in the right coronary sinus (II), the circumflex artery arising from the proximal right coronary artery coursing behind the aorta and anterior to the atria to the left (D), and the left anterior descending artery arising from the proximal right coronary artery travelling from the right side anterior to the right ventricular outflow tract and coursed to the anterior interventricular groove continuing distally and proximally as a normal left anterior descending artery. Such a pattern was already included in the classification but never reported at that time [1]. After a careful revision of the literature, only six cases have been reported with a similar anomalous coronary origin and pattern (Table 1). Only two out of six patients underwent surgical coronary revascularization. In one of them the diagnosis was made postmortem [6]. In our case the aberrant vessel arising from the right coronary artery coursed anteriorly the right ventricular outflow tract as in the reported cases. When the aberrant vessel reached the anterior interventricular groove it continued as a left anterior descending artery at its mid portion which then continued distally as the distal left anterior descending artery and proximally as a proximal left anterior descending artery, having the shape of a “T vessel”. The “T-vessel” configuration has never been reported in the literature.

**Table 1:** Literature review (cases with single ostium from the right sinus, anterior course of the left anterior descending artery, and circumflex artery originating from the right coronary artery)

Author/Reference	Year	Sex	Age	Diagnosis	Stenosis	Treatment
1. Neil *et al*. [7]	2000	Male	48	Postmortem	No	Conservative
2. Smedema *et al*. [6]	2009	Female	46	CA/CT	No	Conservative
3. Gleeson *et al*. [8]	2009	Male	56	CA/CT	Yes	PTCA
4. Mihl *et al*. [9]	2010	Male	69	CA/CT	Yes	PTCA
5. Ramos *et al*. [10]	2010	Female	72	CA/CT	Yes	PTCA/CABG
6. Present case	2015	Male	74	CA	Yes	CABG

*CA* coronary angiography, *CT* computed tomography, *CABG* coronary artery bypass grafting, *PTCA* percutaneous transluminal coronary angioplasty

Coronary anomalies are usually detected during coronary angiography, but exact course determination and relationships are difficult to visualize. The use of computed tomography allows visualization of the entire course of the coronary artery in a three-dimensional image. This was the diagnostic tool of choice in four patients in the reported cases, furnishing further information to the surgical team.

## Conclusions

The reported case with its specific presentation adds further information on this rare form of anomalous origin of the coronary arteries, representing a first report of the configuration that we name the “T-vessel” of the left anterior descending artery. Diagnosis of the coronary anatomy is very important for the invasive cardiologist and cardiac surgeon in cases with a single coronary ostium, such as our case, so that they can proceed with the invasive or surgical treatment when critical coronary artery disease is present.
